# Reduction in *Musca domestica* fecundity by dsRNA-mediated gene knockdown

**DOI:** 10.1371/journal.pone.0187353

**Published:** 2018-01-17

**Authors:** Neil D. Sanscrainte, Hanayo Arimoto, Christy M. Waits, Lucy Y. Li, Dana Johnson, Chris Geden, James J. Becnel, Alden S. Estep

**Affiliations:** 1 USDA/ARS Center for Medical, Agricultural, and Veterinary Entomology, Gainesville, Florida, United States of America; 2 Naval Medical Research Unit No. 3, Cairo, Egypt; 3 Navy Entomology Center of Excellence, CMAVE Detachment, Gainesville, Florida, United States of America; 4 Lovelace Respiratory Research Institute, Albuquerque, New Mexico, United States of America; University of Cincinnati, UNITED STATES

## Abstract

House flies (*Musca domestica*) are worldwide agricultural pests with estimated control costs at $375 million annually in the U.S. Non-target effects and widespread resistance challenge the efficacy of traditional chemical control. Double stranded RNA (dsRNA) has been suggested as a biopesticide for *M*. *domestica* but a phenotypic response due to the induction of the RNAi pathway has not been demonstrated in adults. In this study female house flies were injected with dsRNA targeting actin-5C or ribosomal protein (RP) transcripts RPL26 and RPS6. Ovaries showed highly reduced provisioning and clutch reductions of 94–99% in RP dsRNA treated flies but not in actin-5C or GFP treated flies. Gene expression levels were significantly and specifically reduced in dsRNA injected groups but remained unchanged in the control dsGFP treated group. Furthermore, injections with an *Aedes aegypti* conspecific dsRNA designed against RPS6 did not impact fecundity, demonstrating species specificity of the RNAi response. Analysis of *M*. *domestica* tissues following RPS6 dsRNA injection showed significant reduction of transcript levels in the head, thorax, and abdomen but increased expression in ovarian tissues. This study demonstrates that exogenous dsRNA is specifically effective and has potential efficacy as a highly specific biocontrol intervention in adult house flies. Further work is required to develop effective methods for delivery of dsRNA to adult flies.

## Introduction

The house fly (*Musca domestica* L.) is a major pest of humans and animals throughout the world, especially in areas near intensive animal agriculture production facilities [1]. Their habits of feeding, vomiting, and defecating on both food and feces of all kinds positions them as important transport vectors of a wide range of human and animal pathogens [2–4]. Fly control costs are estimated at $375 million per year in the U.S. alone [5]. Although biological control options are available [6], chemical control with insecticides remains the most common management tactic. House flies rapidly evolve resistance to new toxicants, with the result that there are almost no registered insecticides that provide satisfactory control in much of the world [7–11].

RNA interference (RNAi) has been proposed as a potential novel strategy to target a variety of pest species with the benefits of increased species-specificity and decreased risk to the environment and non-target species [12,10]. The RNAi pathway in Insecta is markedly different from that in plants or *Caenorhabditis elegans* (for example, RNA-dependant RNA polymerases (RdRps) associated with amplifying the systemic RNAi response are not found in insects), thus complicating the development of effective dsRNA delivery methods. Steady progress has been made, however, against particular agricultural pests like the western corn rootworm and the Colorado potato beetle, both of which respond strongly to double stranded RNA (dsRNA) delivered in food or delivered by consumption of dsRNA expressing plants [13–14]. Progress against dipterans has been much slower and susceptibility varies by species and life stage. In *M*. *domestica*, effective RNAi has been demonstrated only in immature life stages where it has been administered by injection or by feeding naked dsRNA or bacterially expressed dsRNA [15–19]. Effective induction of RNAi by exogenous dsRNA has not been demonstrated in adult *M*. *domestica* and is the focus of this investigation.

## Methods

### *Musca domestica* strain and rearing

*Musca domestica* were from an insecticide-susceptible strain (“Orlando Normal”) that has been maintained by ARS since the 1950’s. Immature flies were reared according to Machtinger et al. [20] on a diet consisting of 6.5 L wheat bran, 500 cm^3^ Calf Manna (Manna Pro LLC, Chesterfield, MO), and 3.8 L water. After the 6 d larval development period, puparia were collected from the medium by floating in tap water.

### dsRNA design and production

Double stranded RNA targeting *M*. *domestica* actin-5C (dsActin-5C), ribosomal protein L26 (dsRPL26) and ribosomal protein S6 (dsRPS6) were produced as previously described for the mosquito *Aedes aegypti* [21]. Template selection and design of efficient dsRNA primers utilized the eRNAi web service [22, http://www.dkfz.de/signaling/e-rnai3/] with standard parameters except for selection of “other organism” (*Musca* data not available for query in the eRNAi tool), amplicon size range 150–250 bp, and primer output to include appended T7 promoter sequences. Accession numbers, primer sequences, and amplicon information are listed in Table 1. An *Aedes aegypti* specific dsRNA (ds*Aa*RPS6) and a dsGFP control was produced by Monsanto Company (St. Louis, MO) and have been described previously [21]. Primers for *M*. *domestica* were synthesized by IDT DNA (Coralville, IA). T7-appended primers were used to amplify the selected regions of actin-5C, RPL26 and RPS6 from *M*. *domestica* cDNA produced from females as described below using Platinum™ Taq (Thermo Fisher Scientific, Waltham, MA) and the manufacturer recommended amplification protocol of 94°C for 3 min, 5 cycles of 94°C for 30 sec, 61°C for 30 sec, 72°C for 30 sec, followed by 30 cycles of 94°C for 30 sec, 64°C for 30 sec, 72°C for 30 sec and a final extension at 72°C for 10 min. The appropriate amplicons were gel purified, verified by sequencing (MacrogenUSA, Rockville, MD) and then used for a second round of amplification to produce T7 template. Template was concentrated and washed 2X with 400 μl of nuclease free water (NFW) in a spin concentrator (Amicon 10K MWCO, Millipore, Billerica, MA). Purified template was reverse transcribed to dsRNA using the MegaScript® kit (Ambion, Thermo Fisher Scientific, Waltham, MA). The manufacturer’s protocol was followed with the following exceptions. The entire kit was used (20 reactions) in one tube with 20 μg of template, reverse transcription was allowed to proceed for 24 hours at 37°C, and after purification the resulting dsRNA was concentrated in a spin concentrator to >12 μg/uL as assessed by NanoDrop™ 2000 (Thermo Fisher Scientific, Waltham, MA). Products were maintained at -20°C until dilution for injection.

**Table 1:** Primers for production of double stranded RNA and qPCR analysis.

Name	Gene accession	Position	Forward primer^a^	Reverse primer^a^	Size (bp)^a^
T7-*Mdom*RPS6	XM_005175415.3	297–459	TGAAACAGGGTGTCCTTAGC	CCCTTCTTGACGATGACCAA	163
q*Mdom*RPS6		82–213	GGTGTTCCTCCAAATCAGACA	ACTTGGCCCATACGCTTCTC	131
T7-*Mdom*RPL26	XM_005177118.3	178–360	AAAGAACCGCAAGCGCCATT	GCTTGGACAACCTTGCCAAC	183
q*Mdom*RPL26		407–493	ATGCCAACGGTACCAACGTT	AGCTTTGCGATCCTTGTCCA	86
T7-*Mdom*Actin-5C	XM_005189193.3^b^	818–974	TTGAACAAGAAATGGCCA	TGGATACCGCAGGCTTCCAT	158
q*Mdom*Actin-5C		657–743	TTGCGTTTGGATTTGGCTGG	GCGGTGGTGGTGAAAGAGTA	86
T7-*Aae*RPS6^c^	AAEL000032	240–391	GTCCTGACCAACACCCGT	CCCTTCTTGACGACGATCAG	152
q*Mdom*RPL24	XM_005176417.3	202–327	CCCAATACCTTAACCGGACG	TGGGTGTGATTTGGACCTTT	126
q*Mdom*Actin-5	XM_005182498.3	97–194	TGCCACGTTCGCATAAACAT	GCAGAGGGTTCATCGTCACA	98

^a^ Primers for double stranded RNA production include the T7 promoter sequence at the 5’ end (TAATACGACTCACTATAGGG). This promoter is not shown or used in size calculations.

^b^ Designed to target all known alternate transcripts (XM_020038818.1 and XM_005189192.3) for this gene.

^c^ Primer sequences found in Estep et al. (2016). dsRNA produced commercially.

### Injection methods

Groups of 50–65 *M*. *domestica* female adults (2–3 d post-emergence) were injected with either dsGFP, dsActin-5C, dsRPL26, dsRPS6, or ds*Aa*RPS6. Microinjections were performed on a Nanoliter 2000 (World Products Inc., Sonoma, CA) using fine-pulled capillary needles according to Estep et al. [21] and modified as follows. House flies were cold anesthetized on ice and females staged ventrally exposed on slides. Flies were injected distal to the right thoracic spiracle with 5 μg of dsRNA construct (483 nL). Flies recovered in cohorts of five in mesh covered cups before inversion onto cotton balls soaked with 10% sucrose, and injected flies had continuous access to 10% sucrose up to 3 d post-injection (PI). All microinjection experiments were replicated three times.

### Oviposition assay/mortality counts

Three days PI, flies were provided with food composed of granulated sucrose, powdered milk, and dried egg yolk in an 8:8:1 ratio (by volume) for an additional 4 d before testing for oviposition (= 7 d PI). Oviposition media was prepared by making a 1:1 mixture of spent fly larval rearing medium and cow manure. This mixture was wrapped in black cotton cloth, moistened, placed in a 60 cm^3^ cup, and put in the cage with flies for 6 h. Eggs were then rinsed off the cloth with water, placed in a graduated conical centrifuge tube, and the volume of eggs determined. Water was added to the eggs to prepare a 1:20 suspension of eggs. Eggs were counted in three 0.5 mL aliquots of the suspension, and the mean was used to estimate the total number of eggs deposited in the cage. Mortality in each cage was monitored daily for 6 d and mean egg deposition per live female calculated for each cage. Complete egg clutches were not collected for species specificity experiments and so relative oviposition output was determined as described below.

### Ovarian morphology

Images of dissected ovaries prior to oviposition from representative samples at 6 d PI were acquired on a Keyence VHX-5000 imaging system (Keyence Corporation of America, Itasca, IL), 50 × magnification. The degree of egg maturation was determined using the six-point scale of Tyndale-Biscoe and Hughes [23], where stage 0 represents oocytes with no visible yolk deposition and V indicates mature eggs with vitellogenesis complete and the chorion in place.

### Sample collection and analysis

#### Target transcript qPCR analysis

From three replicate experiments, five individual flies of each treatment (dsGFP, dsActin-5C, dsRPL26, dsRPS6 and ds*Aa*RPS6) were collected at 3 d PI for expression analysis. Total RNA was isolated from each individual adult female as described below and analyzed for expression of actin-5C, RPL26 and RPS6.

Relative expression (RE) of RPS6 was also assessed in dissected tissues. Cohorts of five flies from two injection treatments (dsGFP and dsRPS6) were dissected after 4 d of exposure to a protein source (= 7 d PI), and each fly was separated into the three major body segments on a BioQuip chill table (Model 1614, Rancho Dominquez, CA). Abdomens were subsequently teased open and ovaries were removed with as little additional tissue as possible. Each tissue type (head, thorax, abdomen (with ovaries removed), ovaries) from the five flies was pooled to produce one sample for subsequent RNA isolation. Dissections were completed for flies from each of the three biological replicates.

#### RNA/cDNA preparation

Total RNA was isolated from individual whole adult house flies and tissues using the Quick-RNA™ MiniPrep kit or ZR-Duet™ DNA/RNA MiniPrep kit (Zymo Research Corp, Irvine, CA) as per manufacturer’s protocols with the addition of an on-column DNase I step to ensure no contaminating DNA was present in downstream qPCR. Quantification and purity was assessed on a Nanodrop™ 2000 (Thermo Fisher Scientific, Waltham, MA) with A_260_ /A_280_ routinely >1.95. The cloned AMV First-Strand cDNA Synthesis Kit (Invitrogen, Thermo Fisher Scientific, Waltham, MA) with oligo(dT) primer was used as per manufacturer’s instructions for generating cDNA from 300 ng of total RNA.

#### qPCR

Relative expression of house fly transcripts was measured on a StepOnePlus™ Real-time PCR System using SYBR green chemistry (Applied Biosystems, Thermo Fisher Scientific, Waltham, MA) as previously described (Estep et al. 2016). Briefly, 10 μL qPCR reactions were prepared in duplicate containing 0.2 μM F/R primers, 1X SYBR Select, and 0.5 μL cDNA (equivalent to 7.5 ng of input total RNA). Primers were designed from the transcripts for *M*. *domestica* genes ribosomal protein L24 (XM_005176417.3), ribosomal protein L26 (XM_005177118.3), ribosomal protein S6 (XM_005175415.3), actin-5C (XM_020038818.1, XM_005189192.3 and XM_005189193.3), and actin-5 (XM_005182498.3). Primer sequences and amplicon sizes can be found in Table 1. A common dsGFP injection group was included as a reference sample on each 96-well plate when direct comparison of values across plates was needed. Relative expression was calculated using the Livak method (2^(-ΔΔCt)^) [24].

### Statistical analysis

Oviposition assay data was converted to mean clutch size/fly by dividing the total reproductive output (total number of eggs) by number of flies introduced into the laying chamber. For the species specificity experiments, where complete egg clutches were not collected, percentages for each treatment group were calculated relative to dsGFP. Statistical analysis was performed in SigmaPlot v13 (SyStat Software, San Jose, CA). The null hypothesis is that the treatments were not different. Normality was determined by the Shapiro-Wilk test, and statistical significance by ANOVA and means separation was performed using the Holm-Sidak test. A comparison of mortality occurring during the oviposition assay was performed using a Kruskal-Wallis non-parametric analysis due to the non-normal behavior of the dataset.

Quantitative PCR data were normalized to single (actin-5) or dual reference genes (actin-5 and L24) in accordance with MIQE guidelines. When using two reference genes, a geometric mean Ct was used as the reference value for calculation of ΔΔCt according to standard methods [24]. For expression experiments on whole flies at 3 d PI, a sample from the dsGFP injection group was used as the reference sample for each experiment and was included on each plate to allow plate to plate comparison. Comparisons of RE were analyzed in SigmaPlot by t-test when the data were normally distributed and with the Mann-Whitney rank sum test when data were non-normal.

## Results

### Effects on oviposition and ovarian morphology

**Fig 1:**
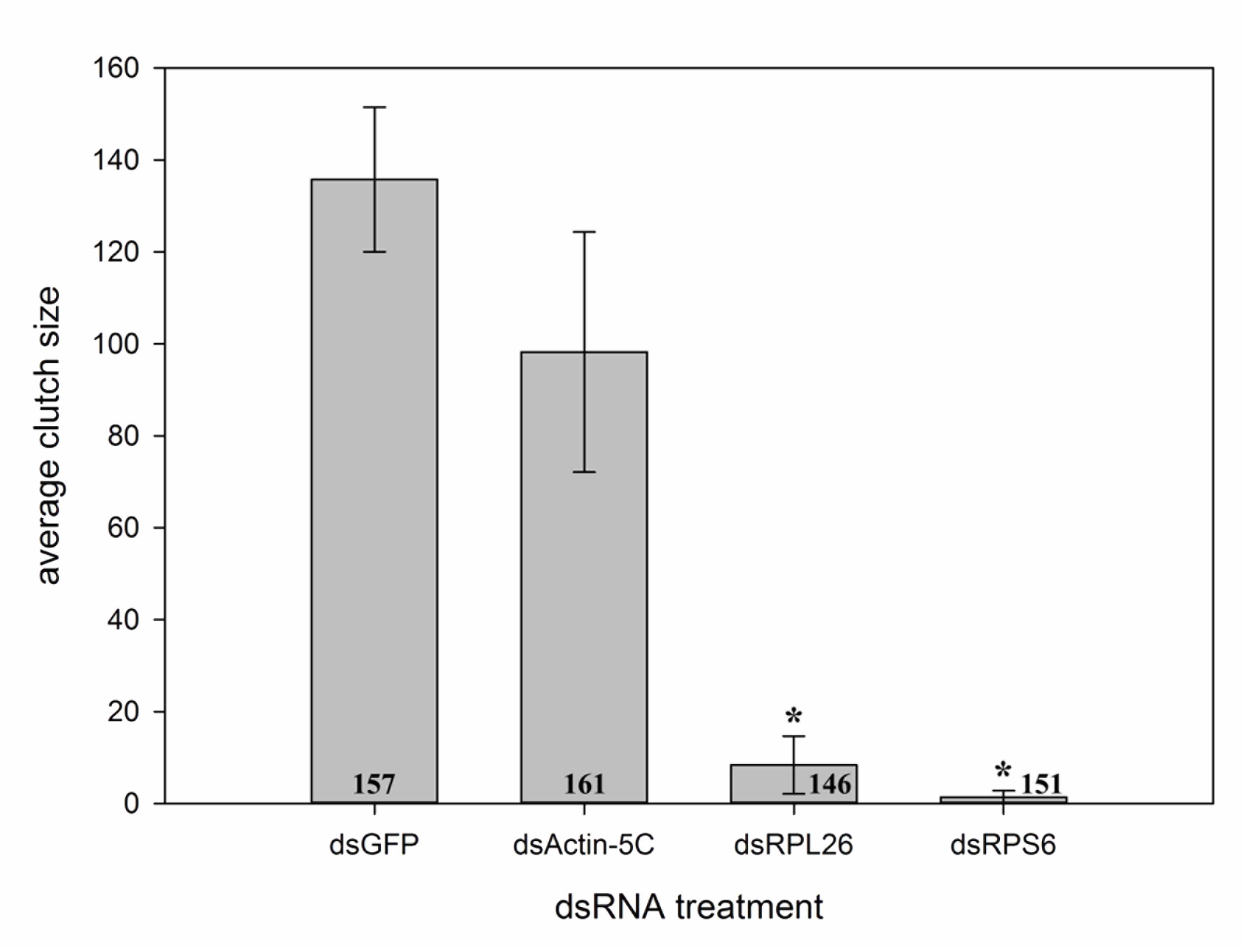
Average clutch size of *M*. *domestica* injected with 5 μg of a non-target control (dsGFP) or dsRNA constructs designed against house fly transcripts (actin-5C, ribosomal protein L26 and ribosomal protein S6). Significant reduction compared to dsGFP was seen 7 days post-injection with dsRPL26 and dsRPS6 treatments. Error bars represent mean±SE and * represents significance at P<0.05. Numbers at the base of columns indicate the total number of individuals injected. Data represents 3 replicate experiments.

*Musca domestica* injected with various dsRNA constructs designed from house fly as well as *Ae*. *aegypti* targets were allowed to oviposit and egg production was quantified. A significant reduction in mean output per female was observed in house flies injected with dsRPL26 (8.4±6.3, mean±SE, df = 2, P = 0.002) and dsRPS6 (1.4±1.4, df = 2, P = 0.002) when compared to flies injected with the control dsGFP (135.7±15.7) (Fig 1). The dsActin-5C treated flies did not show a significant reduction in clutch size (98.2±26.1, df = 2, P = 0.237). Mortality rates were low (average 2.6–14.6%) for all treatments and showed no statistical difference (K-W test, P = 0.947, H = 0.365, df = 3).

**Fig 2:**
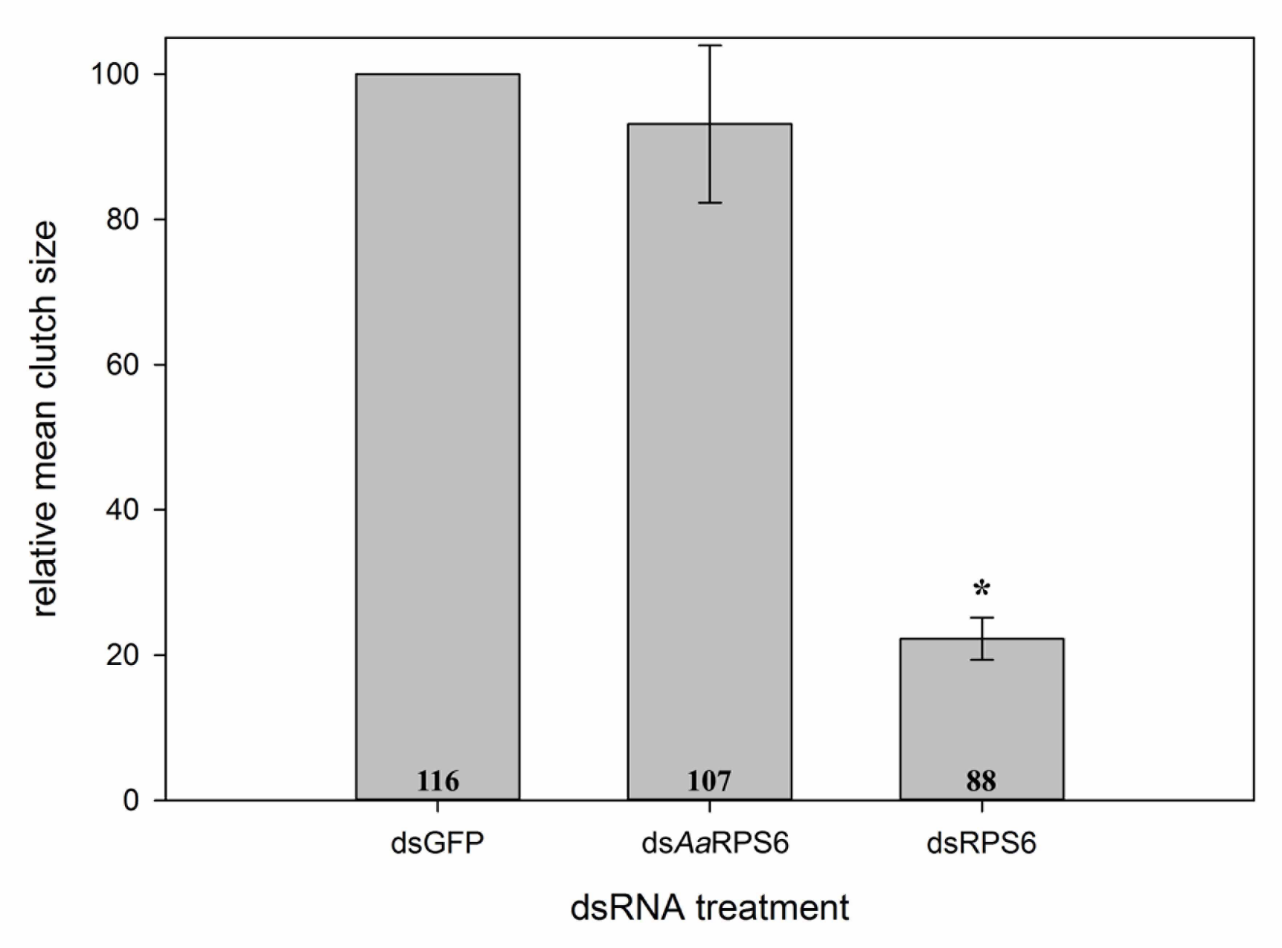
Fecundity in *M*. *domestica* injected with 5 μg of dsRNA constructs designed against *Ae*. *aegypti* RPS6 (ds*Aa*RPS6) and *M*. *domestica* dsRPS6 7 days post-injection. Numbers of eggs laid by the ds*Aa*RPS6 and dsRPS6 treatments were normalized against the control (dsGFP) treatment as partial egg clutches were collected. Significant reduction when compared to dsGFP was seen in the fly dsRPS6 treatment but not in the mosquito ds*Aa*RPS6, thereby showing a species specific RNAi response. Error bars represent mean±SE and * represents significance at P<0.05. Numbers at the base of columns indicate the total number of individuals injected. Data represents 3 replicate experiments.

Injection of dsRNA designed from the *Ae*. *aegypti* RPS6 gene was evaluated in parallel with the *M*. *domestica* dsRPS6. Egg production was not significantly reduced in *M*. *domestica* treated with ds*Aa*RPS6 relative to the control group (dsGFP treatment), whereas *M*. *domestica* injected with dsRPS6 laid significantly fewer eggs (P<0.001) than both the control and ds*Aa*RPS6 treatments (Fig 2).

**Fig 3:**
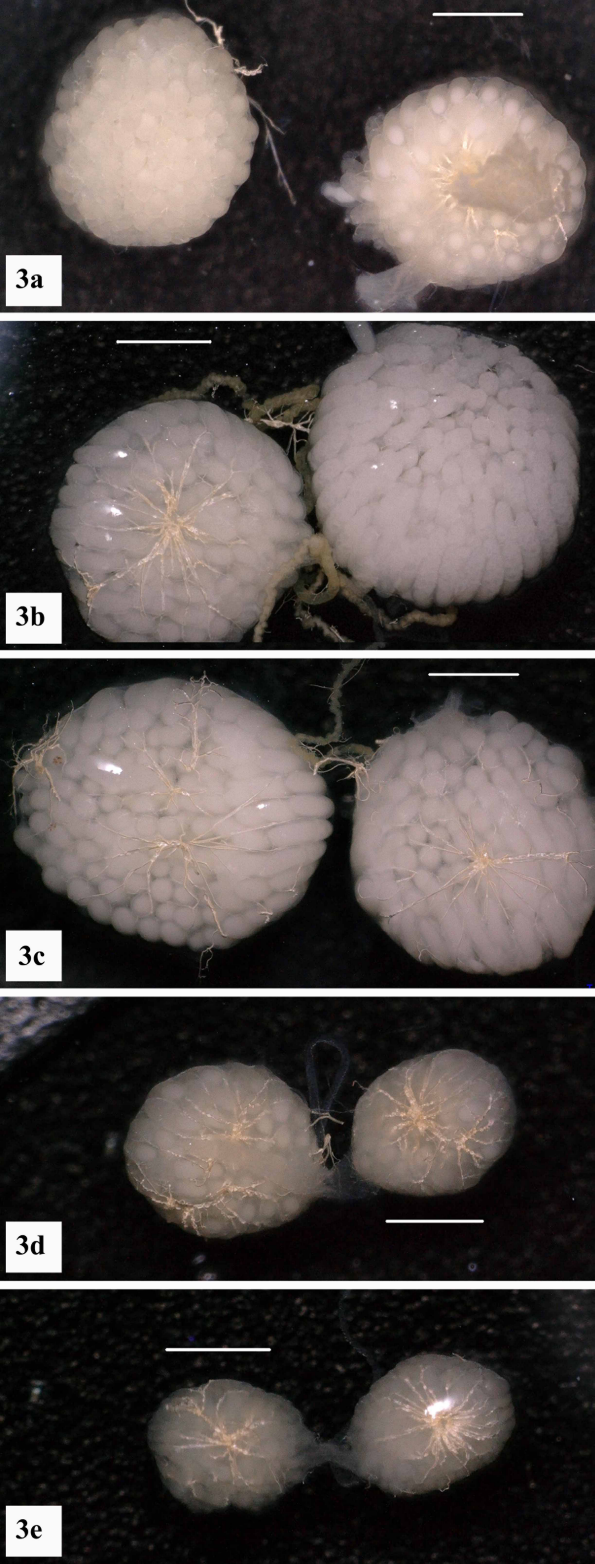
**Ovarian development of untreated *M*. *domestica* (a) as well as individuals injected with a non-target control (dsGFP—b) or dsRNAs targeting house fly actin-5C (c), RPL26 (d), and RPS6 (e).** Dissections were performed after feeding on a protein source for 4 days (6 days post-injection). Untreated, dsGFP and dsActin-5C treated ovaries are all fully gravid and show eggs with high levels of yolk deposition. Ovaries from females injected with dsRPL26 and dsRPS6 show poorly developed eggs which resulted in significantly reduced fecundity. Micron bars are ~1 mm.

Six days PI and prior to oviposition, ovaries showed substantial vitellogenin deposition in non-injected flies (Fig 3A) and flies injected with dsGFP or dsActin-5C (Fig 3B and 3C respectively). Most of these flies were in ovarian stages IV-V on the Tyndale-Biscoe [23] scale (Fig 3A–3C, S1 File). Markedly reduced development is evident in ovaries from individual flies injected with either dsRPL26 (Fig 3D) or dsRPS6 (Fig 3E). Ovaries from these flies ranged from stages I-III, indicating incomplete vitellogensis (Fig 3D and 3E, S1 File). Ovarian morphology was visually similar between the dsRPL26 and dsRPS6 treatment groups (Fig 3D and 3E).

### RNAi mediated gene knockdown

**Fig 4:**
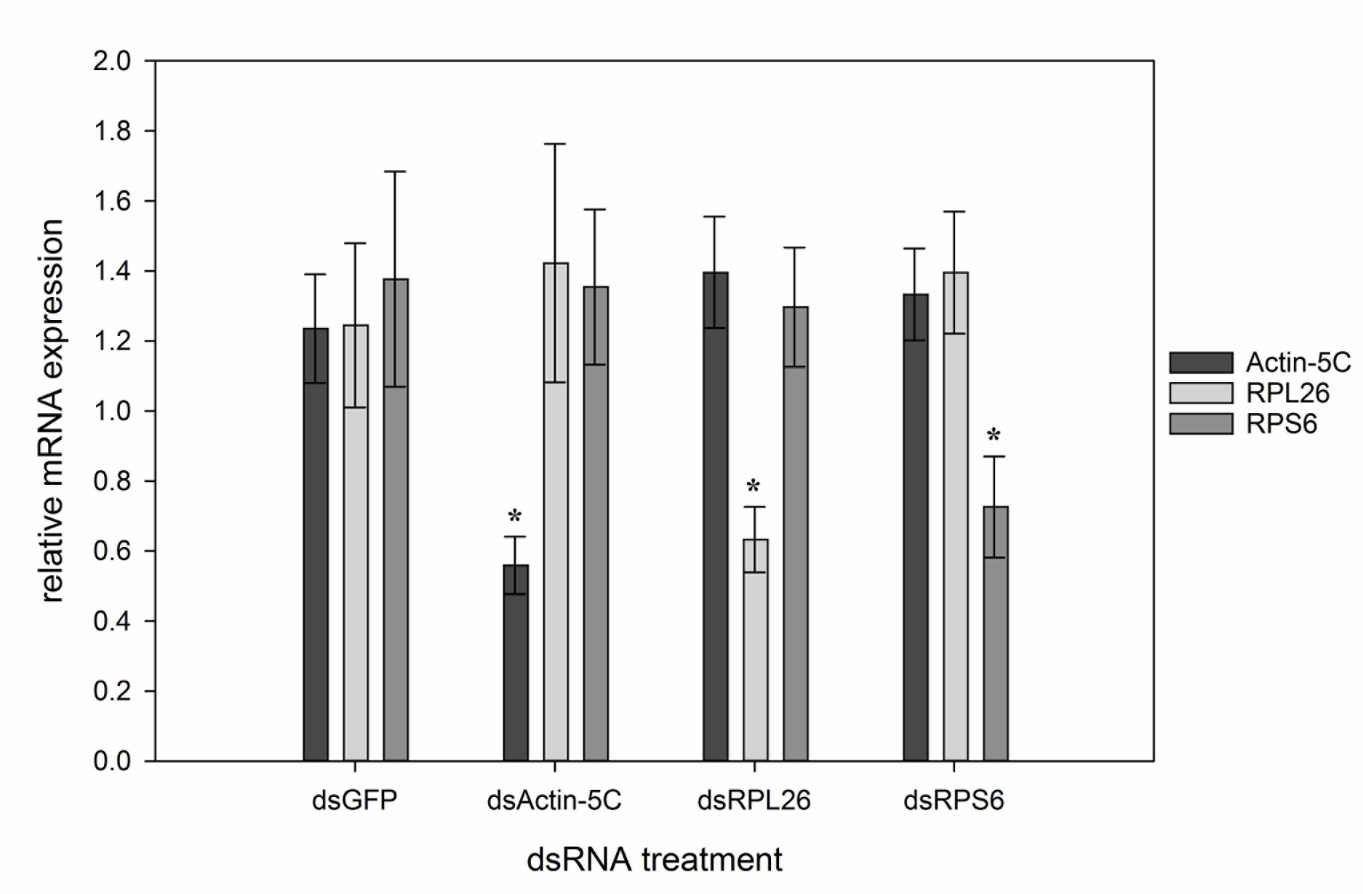
Average relative expression (RE) of three transcripts in individual whole female *M*. *domestica* injected with 5 μg of dsGFP, dsActin-5C, dsRPL26 and dsRPS6. Individual expression levels were all normalized to a single dsGFP sample for each replicate. RE for actin-5C, RPL26 and RPS6 were all significantly lower in their respective dsRNA treatment groups when compared to RE in dsGFP treated house flies. Non-target transcripts remained at normal expression levels in all treatment groups indicating a clear RNAi response to exogenous dsRNA. Data represents n = 15 flies total from 3 replicate experiments except dsRPS6 (n = 14).

We examined the relative expression levels of the targeted transcripts in individual female flies that were injected with the control dsGFP or *M*. *domestica* specific dsRNA sequences for actin-5C, RPL26 and RPS6. Three days PI, transcript expression was significantly reduced in all three *M*. *domestica* dsRNA treatments (dsActin-5C, P<0.001; dsRPL26, P = 0.006; dsRPS6, P = 0.022) when compared to dsGFP injected flies (Fig 4). Expression levels of genes not targeted by a specific dsRNA treatment did not show reduced expression relative to dsGFP, indicating the specific action of the dsRNA triggers on the targeted transcripts. The transcript level of actin-5C was significantly reduced after dsActin-5C injection even though there was no significant reduction in fecundity observed in the oviposition assay or ovarian morphology.

**Fig 5:**
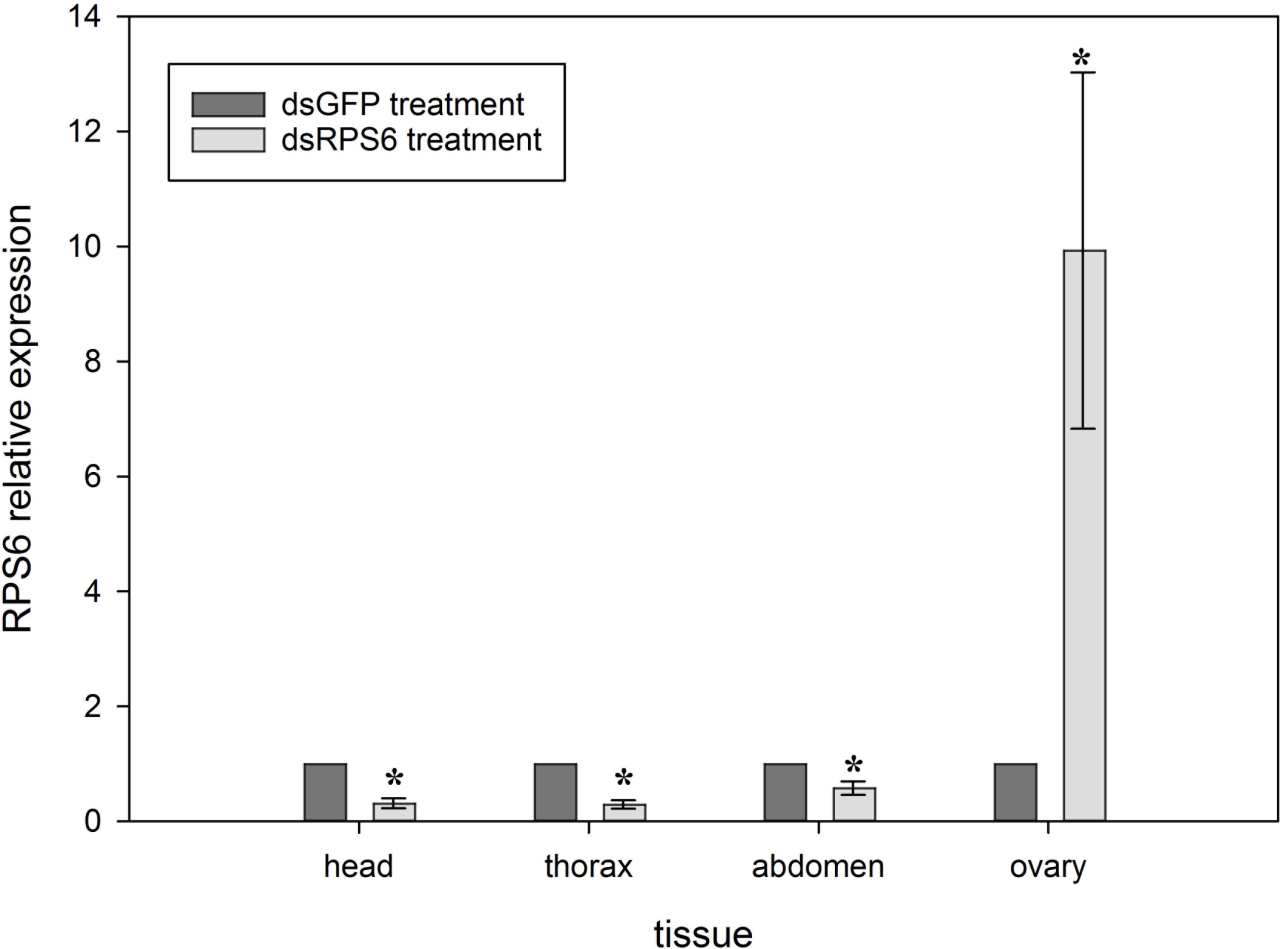
Average relative expression (RE) of RPS6 in tissues from female *M*. *domestica* injected with 5 μg of dsGFP or dsRPS6. All values are normalized to RE levels from flies injected with dsGFP. Significant reduction (P<0.05) in RPS6 expression compared to the dsGFP control group was observed in the head, thorax, and abdomen for the dsRPS6 treatment. A significant increase in RPS6 expression was observed in ovarian tissues from dsRPS6 treatments when compared to dsGFP injected samples. Error bars represent mean±SE and * represents significance at P<0.05. Tissues samples were pooled from 5 individual flies and injections were replicated 3 times.

Ribosomal protein S6 expression levels measured in dissected *M*. *domestica* tissues 4 d after exposure to a protein source are shown in Fig 5. When normalized to tissue samples injected with dsGFP, dsRPS6 injected samples had RPS6 levels significantly reduced in the head (P = 0.001), thorax (P<0.001) and abdomen (P = 0.023). Significant upregulation of RPS6 expression (P = 0.045) was observed in ovaries from dsRPS6 injected flies compared to that of dsGFP injections.

## Discussion

Exogenously introduced dsRNA that specifically targets a species and critical transcripts is an emerging technology that is being developed for the control of the western corn rootworm and the Colorado potato beetle [13,25]. While coleopteran pests appear particularly sensitive to this new technology, numerous studies have been conducted with variable success against pest species of Lepidoptera, Diptera, Hemiptera, Hymenoptera, Isoptera, Orthoptera and Blattaria [26,27]. In addition to insects, numerous human RNA-based pharmaceuticals are close to market [28].

Because *M*. *domestica* are an important pest, we examined the feasibility of using species-specific nucleic acid based products to control the adult life stage. We examined two of the critical elements required for effective control by RNAi. The literature indicates that the various life stages of insects are not equally susceptible to dsRNA [29–31]. Currently, in *M*. *domestica*, effective RNAi has only been demonstrated by injection into the embryo that is carried over into the larval stages of the fly [15–19]. In this study we clearly show, for the first time in adult *M*. *domestica*, that expression of three different targets can be specifically reduced with injected dsRNA. In whole organism preparations, the specificity of this knockdown was limited to the transcript of interest and was not observed to be a general phenomenon from introduction of dsRNA. Injection of a conspecific dsRNA from *Ae*. *aegypti* (76% sequence similarity to the muscoid dsRPS6) did not produce a reduction in oviposition in *M*. *domestica*. This indicates a species-specific response to the muscoid dsRPS6 likely due to activation of the RNAi pathway. Based on *in silico* comparison, only two of the 131 possible 21-mer siRNAs from ds*Aa*RPS6 would have two mismatches but more than 90% would have four or more mismatches and would not be expected to be effective [21].

Examining tissue specific knockdown from injection of dsRPS6 did not demonstrate the same pattern that was observed in *Ae*. *aegypti* where knockdown was strong in the fat body of the abdomen and the ovary [21]. In *M*. *domestica*, levels of transcript were significantly higher in the ovaries (in comparison to dsGFP treatment) after injection of dsRPS6. While we are confident that we consistently observed overexpression of RPS6 in the ovaries and were not observing an artifact created by shifting expression in our control genes (S1 File), the result seems counterintuitive. Several possible explanations exist. First, upregulation has been described in response to dsRNA uptake in insects or vertebrates due to activation of various immune pathways [32–35] so our observation may be similar. A second possibility is that the ovaries’ role in signaling the oogenesis process and as the ultimate destination for vitellogenins produced in the fat body, but not as the site of vitellogenin production itself [36], may make the ovarian RPS6 or RPL26 transcript levels irrelevant. Thus, knockdown of RPS6 and RPL26 elsewhere (as we observed) may be much more important. It is also possible that interference in oogenesis signaling by RPS6 knockdown may cause changes in the cell type and composition of the ovaries themselves [37]. Further work to understand whether the upregulation was a general non-specific phenomenon or target-specific (based on the sequence of the dsRNA) needs to be conducted, but based on the data relative to dsGFP treated flies there does appear to be an element of specific response.

Another critical element for potential use of nucleic acid based control methods against *M*. *domestica* is to identify effective targets that impact host survival. Direct mortality shortly after treatment is desirable, however, this has been difficult to produce consistently in adult dipterans for reasons that are unclear even with knockdown of the targeted transcript [38]. However, dsRNA-mediated targeting of various ribosomal transcripts critical for fecundity has been demonstrated in mosquitoes and mites [21,39–40] Knockdown of transcripts for RPS3a has shown a similar effect in *Culex* mosquitoes [39]. Based on these previous reports, we targeted two ribosomal protein transcripts (RPL26 and RPS6) and the transcript for actin-5C in *M*. *domestica* adults. We identified two effective dsRNA triggers (dsRPL26 and dsRPS6) that reduce fecundity. In contrast, we did not observe fecundity reductions when targeting actin-5C even though it had similar levels of reduced relative expression when assessed by qPCR.

A third critical element for an effective RNA based product is an efficient delivery method that is practical for field application. Thus far, effective delivery methods for adult dipterans is lacking. Oral uptake of dsRNA has been demonstrated but the resulting effect on transcript levels is very limited and no specific phenotype was induced [41–42]. Early studies indicated that the mechanism of oral uptake of RNA (in the form of RNA viruses) was relatively inefficient in carbohydrate-containing solutions when compared to uptake from a blood containing solution [43]. In this study, we do not address the issue of delivery but instead focus on demonstrating that RNAi mediated gene knockdown can be effective in adult house flies.

Future work will address validating nucleic acid based products as a potential control method for house flies through successful delivery methods. Determining the effective period for this knockdown response is important; a permanent loss of fecundity would certainly be more useful as a control tool than a transient loss. Knockdown of RPS6 in mosquitoes was effective through multiple gonotrophic cycles so we speculate that it may also be relatively long lasting in the fly as the level of transcript reduction and ovarian morphology appear comparable [21]. Discovery of other dsRNA triggers that induce mortality would be valuable for suppression of house fly populations. While this study has shown that the RNAi pathway can be used to knockdown transcripts in adult *M*. *domestica* and that some dsRNAs targeting ribosomal transcripts can cause reduced fecundity, the major challenge remains delivery of the dsRNA trigger to the adult. RNAi has been demonstrated against several targets in larval *M*. *domestica* and it may be that delivery is easier to the larva than the adult but this is as of now unknown. Thus, it should be determined whether the phenotype we induced here can be induced in immature life stages and persist into the adult life stage. Until effective delivery methods are developed for adult flies, triggering the RNAi pathway is of use only as a tool to study functional expression.

## Supporting information

S1 File Raw data and additional photos of ovarian dissections. (XLSX)
